# Effects of supplementation with n-3 polyunsaturated fatty acids on cognitive performance and cardiometabolic risk markers in healthy 51 to 72 years old subjects: a randomized controlled cross-over study

**DOI:** 10.1186/1475-2891-11-99

**Published:** 2012-11-22

**Authors:** Anne Nilsson, Karl Radeborg, Ilkka Salo, Inger Björck

**Affiliations:** 1 Division of Applied Nutrition and Food Chemistry, Department of Food Technology, Engineering and Nutrition, Lund University, Lund, Sweden; 2 Department of Psychology, Lund University, Lund, Sweden

**Keywords:** Omega-3 PUFA, DHA, EPA, Fish oil, Dietary prevention, Cognitive performance, Working memory, Metabolic disorders, Ageing

## Abstract

**Background:**

Higher plasma n-3 polyunsaturated fatty acids (PUFA) have been associated with a lower risk of age related cognitive decline, and to beneficially affect cardiometabolic risk factors. A relation exists between metabolic disorders such as diabetes type 2 and cognitive decline. Results regarding the potential effects of n-3 PUFA on risk factors in healthy subjects are divergent, and studies regarding the possible relation between cardiometabolic parameters and cognitive performance are scarce. The objective was to evaluate the effects of five weeks intake of long chain n-3 PUFA on cognitive performance in healthy individuals, and to exploit the possible relation between outcomes in cognitive tests to cardiometabolic risk parameters.

**Methods:**

Fish oil n-3 PUFA (3g daily) were consumed during 5weeks separated by a 5 week washout period in a cross-over placebo controlled study, including 40 healthy middle aged to elderly subjects. Cognitive performance was determined by tests measuring working memory (WM) and selective attention.

**Results:**

Supplementation with n-3 PUFA resulted in better performance in the WM-test compared with placebo (*p* < 0.05). In contrast to placebo, n-3 PUFA lowered plasma triacylglycerides (P < 0.05) and systolic blood pressure (*p* < 0.0001). Systolic blood pressure (*p* < 0.05), f-glucose (*p* = 0.05), and s-TNF-α (*p* = 0.05), were inversely related to the performance in cognitive tests.

**Conclusions:**

Intake of n-3 PUFA improved cognitive performance in healthy subjects after five weeks compared with placebo. In addition, inverse relations were obtained between cardiometabolic risk factors and cognitive performance, indicating a potential of dietary prevention strategies to delay onset of metabolic disorders and associated cognitive decline.

## Background

Long chain n-3 polyunsaturated fatty acids (n-3 PUFA, docosahexaenoic acid (DHA) and eicosapentaenoic acid (EPA)) are important for optimal brain function and mental health [1,2]. In prospective cohort- [3] and cross-sectional studies [4] of middle aged and elderly populations, higher proportions of n-3 PUFA in plasma were linked to a lower risk of cognitive decline. A number of studies further reveal that higher fish consumption promoted less decline and better cognitive functions [5-9]. However, controlled intervention trials of the effect of n-3 PUFA on cognitive functions in healthy subjects are scarce, and results from the limited number of studies available are divergent. A double-blind, placebo-controlled trial in 302 cognitively healthy subjects (65 years) revealed no effects on cognitive domains of attention, sensorimotor speed, memory, and executive function after 26 weeks supplementation with 1800 mg/d or 400 mg/d EPA+DHA [10]. Contrary, a randomized, double-blind, placebo-controlled trial in 458 healthy subjects resulted in beneficial effects on a visuospatial learning and episodic memory test after 24 weeks supplementation with 900 mg daily intake of DHA [11]. In a smaller study, 33 subjects received a diet supplemented with n-3 PUFA from fish oil (daily, 1.6g EPA + 0.8g DHA) for 35 days, whereas 16 subjects instead were supplemented with olive oil (placebo) [12]. Supplementation with n-3 PUFA was associated with improved attentional functions and mood. In another randomized double-blind intervention study, fifty-four healthy young adults were received either n-3 PUFA supplementation (daily 2.3 g of n-3 PUFA; 1.74 g EPA, 0.25 g DHA) or placebo (olive oil) for 4 weeks [13]. PUFA supplementation was associated with performing fewer risk-averse decisions.

Cardiometabolic disorders such as the metabolic syndrome, impaired glucose tolerance, and diabetes are associated with higher risk of cognitive decline, e.g. decrease in memory and executive functioning [14-16], information processing speed, attention [17], and overall intellectual functioning [18,19]. Long chain n-3 PUFA intervention studies have shown benefits on several key metabolic risk factors, e.g. lowers the blood pressure and triglycerides [20], reduce inflammatory markers [21], and improve glucose metabolism [22] and insulin sensitivity [23].

The present study undertakes to evaluate the effects of dietary supplementation with n-3 PUFA on cognitive performance in healthy individuals; and to relate cognitive outcome to cardiometabolic risk parameters. For this purpose, healthy middle aged to elderly subjects (51–72 years) with BMI 20–30 kg/m^2^ were provided n-3 PUFA supplement from fish oil (3 g/day) or placebo for five weeks, respectively, in a randomized cross-over design with a five-week wash-out period. Cognitive tests of working memory and selective attention were executed after the n-3 PUFA- and placebo periods, respectively, and metabolic risk markers were determined in blood prior to and after the PUFA- and placebo periods.

## Subjects and methods

### Ethics statement

This study was conducted in compliance with the guidelines laid down in the Declaration of Helsinki (ethical principles for research involving human subjects). All procedures involving human subjects were approved by the Regional Ethical Review Board in Lund, Sweden (protocol 2008/5). Written informed consent was obtained from all subjects.

### Study population

Volunteers, 30 women and 14 men aged 51–72 years (mean ± SD: 63 ± 5 years) with BMI between 20–30 kg/m^2^ (mean ± SD: 25 ± 3) from the south of Sweden, were recruited to the study through advertisement in local newspapers. Exclusion criteria were blood glucose > 6.1 mmol/L, BMI > 30 kg/m^2^ and known metabolic diseases, gastro-intestinal disorders or known cognitive decline. Medication for high blood pressure was allowed, but had to be kept constant during the study period. The subjects had to be fluent in the Swedish language. All but one subject (born in UK, but fluent in the Swedish language) were native Swedish.

Recruitment began October 2008. Trials took place between January 2009 and June 2009. Out of the forty-four volunteers enrolled in the study, four dropped out after the first intervention period (two after the placebo period and two after omega-3); three due to personal reasons and one was excluded due to reporting suffering from a disorder affected wakefulness. Forty subjects completed both intervention periods, but two subjects were excluded due to detection of abnormal fasting blood glucose concentrations (7.0 and 6.7 mmol/L, respectively). In total, results from 38 completers (28 women and 10 men) aged 51–72 years (mean 63.3 ± 5.3) and with BMI between 20–31 kg/m^2^ (mean 25.0 ± 2.8) were evaluated. Twenty out of the 38 subjects started with n-3 PUFA and 18 with placebo. Twenty-two subjects were senior citizens. One of the participants was an occasional smoker, but didn’t smoke the day before or during the test days. The participants were interviewed regarding eating habits and health status prior to the onset of the study. In addition, at each visit, i.e. prior to and after each intervention period, a questionnaire regarding current diet, physical activity, health status and use of medicine, were filled in. All participants consumed an ordinary Swedish diet, including meat, and fish every week. The subjects were told to continue with their habitual diet throughout the study period. When comparing diet journals within each subject, it could be stated that there were no major changes in diet or physical activity during the whole study period. No physical examination was included. Based on information from interviews and forms it was revealed that seven participants were medicated for hypertonia, and one subject with cholesterol lowering medication (Simvastatin). One subject was medicated for depression since several years, without any symptoms and changes in medicine for at least twelve month. Three participants suffered from mild osteoarthritis (one was medicated with glucosamine). Post-intervention evaluation of base-line data revealed that five subjects had high levels of triacylglycerides (2.3, 2.3, 2.7, 2.8, and 3.3 mmol/L) and three subjects showed borderline concentrations (1.8, 2.0, and 2.2 mmol/L).

No major side effects or problem to consume the supplements were reported.

### Test product and placebo

#### Test product

The dietary supplement consisted of capsules containing 1000 mg fish oil, whereof 600 mg was n-3 PUFA (EPA 300 mg, DHA 210 mg and 90 mg unspecified) (Pikasol fish oil capsules, Axellus VS, ORKLA, Oslo, Norway). Five capsules per day were consumed; resulting in a total daily intake of 3 g n-3 PUFA (EPA 1500 mg, DHA 1050 mg and 450 unspecified).

#### Placebo

The placebo supplement was provided as two tablets per day containing in total 366 mg dicalcium phosphate (E 341), 150 mg microcrystalline cellulose (E 460) and 4 mg magnesium salts of fatty acids (E 470b). The placebo was provided by Axellus VS, ORKLA, Oslo, Norway.

### Study design

The study had a cross-over, randomised but balanced design. Out of the forty-four subjects described above, forty completed both intervention periods. Twenty subjects (14 women and 6 men) started with five weeks daily consumption of omega-3 PUFA, and consumed placebo in a second five weeks intervention period, and 20 subjects (14 women and 6 men) were enrolled to start with five weeks consumption of the placebo. The subjects visited the experimental department at four occasions; in the mornings prior to start of each intervention periods, and in the mornings after finishing the PUFA- and placebo period, respectively.

### Protocol

The evening prior to attendance, at 9.00 pm. the test subjects ate a standardized meal, consisting of white wheat bread with optional spread, and had coffee, tea or water to drink. Thereafter they were fasting until the arrival at the research department. At 07.45 am, the test subjects were weighed and seated to rest for a minimum of 10 minutes before the blood pressure was registered and fasting blood samples collected. A standardized breakfast was served consisting of white wheat bread (Dollar Storfranska, Lockarps bakery, Malmö, Sweden) and apricot marmalade (Ica, Sweden) corresponding to in total 55 g available carbohydrates. Water, 250 ml, or a plain cup of decaffeinated coffee or tea was served with the bread. The breakfast was consumed within 15 min. Thereafter, cognitive tests were performed and capillary blood tests were collected repeatedly up to 180 min post commencing the breakfast. The occupation during in-between the cognitive tests were standardized such that the subjects performed Sudoku.

#### Setting

The study was performed at the division of Applied Nutrition and Food Chemistry, Department of Food Technology, Engineering and Nutrition, Lund University, Sweden.

### Cognitive tests

Prior to the intervention periods, i.e. at visit no. 1 and 3, the subjects performed pilot versions of the cognitive tests to reduce learning effects and stress at the cognitive test days. Measurements of cognitive performance were performed after completion of each intervention period, i.e. at visit no. 2 and 4.

#### Working memory (WM) test

The tests employed in the present study represent an extension of the methodology developed by Radeborg et al. [24]. There are several reasons for choosing WM as a measure of cognitive performance in this study. WM can be defined as a system responsible for simultaneous temporary short term storing and processing of information, and is involved in many everyday activities; such as mathematical problem solving where one often has to remember part of the result in a calculation while performing further mathematical operations. WM represent a fundamental ability for higher-level cognitive processes. Thus, measures of WM capacity have been shown to correlate significantly with activities as diverse as e.g. reading comprehension [25], note taking [26], the following of directions [27], reasoning [28], and complex learning [29]. Some authors [30,31], even claim that WM and general problem solving ability or intelligence, as measured by e.g. Raven´s Matrices, reflect nearly identical constructs. However, whereas intelligence tests generally only can be administered once due to risk of considerable learning effects, WM can be measured repeatedly. In total, three oral WM-tests were included at each experimental day (performed at 60, 110, and 160 min). Two of the tests (at 60 and 160 min) were executed principally as described previously [32], modified by including 12 sets of 3–5 short declarative sentences (four of each number) instead of 4 sentences in all sets. As previously, the sentences could be either semantically meaningful of the type ‘the boy brushed his teeth’, or nonsensical, such as ‘the rabbit struck the idea’. The test leader was blind to the product provided to the test subjects. The sentences were read one by one to the subjects. Immediately after a sentence, he/she had to indicate if it was a semantically meaningful sentence or not. After each set of sentences the subjects had to repeat, in any order, the first noun in each of the sentences. One test could at maximum generate 48 credits. The tests consisted of equal number of sentences that were semantically meaningful (24 credits) and nonsensical (24 credits). It has been described that remembering of a noun in a semantically nonsensical sentence is more demanding [24]. The WM-test could therefore be divided into two parts differing in degree of difficulty. The third WM-test was performed at 110 min and was similar to the tests just described, with the exception of that instead of short sentences, the test was composed of simple additions of two single digit numbers. The test leader presented orally the two figures to be added, and the test subjects were supposed to immediately give an oral answer to the addition. After a set of 3–5 additions, the subjects had to repeat the first figure in each addition. The test could at maximum generate 48 credits. One WM-test took approximately 8–10 min to perform. Four different but comparable WM-tests composed of sentences and two different but comparable WM-tests composed of figures were included in the study.

#### Selective attention (SA) test

The test was based on spatial perception and primarily measured the ability to sustain a prolonged attention, and to control and split the attention to the entire picture on a computer screen. Like the WM-test, the test also dealt with simultaneous temporary storing and processing of information (WM capacity). The storing time required was however shorter compared with the WM-test, whereas the time pressure was higher. The SA was measured using a computerized test made up of 96 pictures, each shown for two seconds on the computer screen. The pictures consisted of a square on a white background, divided into four equally sized smaller squares. One of the smaller squares was red, one square was green, and two squares were uncolored (white), resulting in a total of 12 unique picture combinations. The subjects had to remember the positions of the colored squares, and to compare each new picture that emerged on the screen with the preceding one. Each time a new picture emerged, either the green, the red, or none of the colored squares were in the same position compared with the previous picture. Within the two seconds each picture was shown, the subjects were supposed to indicate by pressing one of three different keys on the keyboard, which of the three possible alternatives that occurred for each new picture. The test began with a short training session, and took approximately 10 min to perform. The test was scored with the number of correct responses (CR, total 95 credits) and for the reaction time (RT) needed to give the answer (i.e. press one of the keys).

### Metabolic risk markers

Physiological test variables were determined prior to and after completing each intervention period. Blood pressure was determined with an automatic blood pressure cuff (Digital Automatic Blood Pressure Monitor, Model M3 Intelligence, OMRON HEALTHCARE CO., LTD, Kyoto, Japan). Finger-prick capillary blood was withdrawn at fasting and at 15, 30, 45, 60, 90, 125, 160 and 180 min after the start of the standardized breakfast for determination of glucose concentrations and glucose tolerance (HemoCue^®^B-glucose, HemoCue AB, Ängelholm, Sweden). Venous blood was withdrawn for determination of fasting levels of serum (s) insulin, s-TNF-α, s-adiponectin, s-free fatty acids (s-FFA), s-triacylglycerol, and plasma (p) malonedialdehyd (MDA). The venous blood samples were centrifuged and plasma and serum separated and stored in a freezer (−40°C) until analyzed.

Methods for analyses of insulin, FFA, adiponectin and triacylglycerols are described elsewhere [33]. S-TNF-α was determined with a sandwich enzyme immunoassay kit (TNF-α ELISA Kit, Immunodiagnostik AG, Germany). Plasma MDA was determined by measure of lipid peroxidation as TBARS as is described in [34], modified by excluding the n-butanol.

### Calculations and statistical methods

Primary outcome measure was results in the WM-test. The sample size was calculated based on a study in healthy middle aged subject, including a similar WM-test as was used in the current study [24]. A significant effect (*p* < 0.05) was detected on WM, with an effect size (Cohen´s d) of *d* = 0.75. In the present study we assumed a smaller effect (*d* = 0.50), resulting in a power of 0.86 in a one tailed statistical hypothesis test, and a power of 0.77 in a two tailed test. In a power calculation, based on table 9–9 and 9–10 in Aron and Aron 82003: Statistics for Psychology, 33 subjects would be enough to get a 80% power. However, to have the possibillity to have a balanced design (balansed with respect to order of products and order of test sequenses), we decided to involve 40 subjects.

The results are expressed as means ± SEM. The influences of the test- and placebo products on the cognitive tests were analyzed by repeated measures ANOVA at the test points, with order of test meals and test meals as independent variables and performance on cognitive tests as dependent variables. Statistical calculations were performed in Stat View 5.0 and SuperAnova 1.11. Treatment effects on physiological test parameters (based on changes from baseline in the intervention -and placebo periods, respectively) were assessed with analysis of variance (ANOVA general linear model) in MINITAB Statistical Software (release 13.32; Minitab inc., State College. PA. USA). Time effects on cognitive tests were assessed with analysis of variance (ANOVA general linear model) followed by Tukey’s pairwise multiple comparison method for means (adjusted means were reported) in MINITAB. Participants acted as their own control. GraphPad Prism (version 4.03; GraphPad Software, San Diego, CA, USA) was applied for calculation of blood glucose incremental areas under the curves (IAUC). Blood glucose IAUC (0–90 min) was used as an estimate of glucose tolerance. Pearson correlations were applied to study relations between physiological test parameters and results in the cognitive tests. Values of *P* ≤ 0.05 were considered statistically significant. Cohen’s *d* is presented to report effect size for significant results [35]. n= 38.

## Results

### Cognitive tests

#### WM-tests

The outcomes from the WM-tests are presented in Table 1. Five weeks dietary supplement with n-3 PUFA from fish oil improved performance in the WM-test at 60 min compared with the placebo F(1,36) = 4.41, *p* = 0.04, *d* = 0.26. There was a tendency towards improvement after n-3 PUFA in total performances in the WM-tests based on sentences (WM-tests at 60 + 160 min) F(1,36) = 3.43, *p* = 0.07, *d* = 0.20. When including only the most demanding part in the statistical calculations, i.e. the semantically nonsensical sentences, the differences in performance after n-3 PUFA compared with placebo became more substantial: WM-tests at 60 min: F(1,36) = 6.87, *p* = 0.013, *d* = 0.34, and WM-tests at 60 + 160 min: F(1,36) = 6.87, *p* = 0.015, *d* = 0.31, Table 2.

**Table 1:** **Results in the WM-tests following five weeks daily dietary supplementation with 3 g omega-3 PUFA from fish oil or placebo, respectively**^**1**^

**Treatments**		
WM-tests ^2^(max 48 credits)	Omega-3	Placebo
60 min	31.4±0.8^a^	30.0±1.0^b^
110 min	33.8±1.4^a^	33.3±1.4^a^
160 min	30.4±0.9^a^	29.6±1.0^a^

^1^Data are given as means per treatment ± SEM, *n* = 38, but only 37 subjects performed the WM-test at 110 min (19 subjects started with placebo and 18 subjects started with PUFA) due to one subjects did not perform the test in time. Labeled means in a row without a common letter differ, *p* < 0.05 (ANOVA).

^2^ At 60 min and 160 min the subjects were supposed to recall nouns and at 110 min the subjects were supposed to recall figures.

**Table 2:** **Results in the most demanding part of the WM-tests following five weeks daily dietary supplementation with 3 g omega-3 PUFA from fish oil or placebo, respectively**^**1**^

**Treatments**		
WM-test (max 24 credits)	Omega-3	Placebo
60 min^2^	14.8±0.6^a^	13.7±0.5^b^
160 min	14.5±0.5^a^	13.8±0.5^a^
Total (60+160 min)^3^	29.3±0.9^a^	27.5±1.0^b^

^1^The data shows results for the most demanding part of the tests, i.e. recall of a noun in semantically nonsensical sentences. Data are given as means per treatment ± SEM, *n* = 38. Labeled means in a row without a common letter differ, ^2^*p* = 0.01, ^3^*p* = 0.015 (ANOVA).

There were no differences in the performance of the WM-tests depending on the consumption sequence of the test product (total word retrieval: *P*=0.85, figure retrieval *P*=0.45). However, there was a [treatment*consumption sequence] interaction in the WM-tests, total word retrieval F(1,36 )= 5.86, *p* = 0.021 and figure retrieval F(1,35) = 6.50, *p* = 0.015, that revealed better performance after n-3 PUFA compared with the placebo in the subject group (20 subjects) that had PUFA in the first intervention period (total word retrieval: F(1,19) = 9.05, *p* = 0.007, *d* = 0.37, figure retrieval: F(1,19) = 4.47, *p* = 0.048, *d* = 0.25), whereas there were no significant differences depending on treatment in the 18 subjects that started with placebo (word retrieval: *p* = 0.69, figure retrieval: *p=* 0.29). There were no significant time effects in performance between the WM-tests performed at 60 min and 160 min (*p* = 0.15). The absence of improvement with time indicates that there were no learning effects in the WM-tests.

#### SA-tests

The results from the SA-tests (CR) are displayed in Table 3. Even if not significant, there was a tendency towards better performance after n-3 PUFA supplementation compared with the placebo in the total SA-test (SA-test 1–4, F(1,34) = 3.10, *p* = 0.087, *d* = 0.10). No differences in the performance were found in the SA-tests depending on the consumption sequence (*p =* 0.15) but there were [treatment*consumption sequence] interactions (total SA-test), F(1,34) = 34.08, *p*= 0.0001, with better performance after the placebo F(1,18) = 9.28, *p* = 0.007, *d* = 0.34 or PUFA F(1,17) = 7.40, *p* = 0.015, *d* = 0.29, depending on being consumed in the second intervention period. The improvements in performance from the first to the second test occasion indicate learning effects in the SA-test. In addition, there was also a time effect during the test day, meaning that the subjects performed inferior in the first SA-test (fasting) compared with the other three SA-tests F(3,107) = 16.35, *p* = 0.000. No differences were seen in reaction times depending on n-3 PUFA or placebo (results not shown).

**Table 3:** **Results in the SA-tests following five weeks daily dietary supplementation with 3 g omega-3 from fish oil or a placebo product, respectively**^**1**^

**Treatments**		
SA-test (max 95 credits)	Omega-3	Placebo
Fasting	77.39±2.57	75.28±2.79
45 min	80.84±1.92	79.97±2.29
95 min	81.16±2.02	81.43±1.90
145 min	81.73±1.77	81.62±2.08
Total (0-145min)^2^	322.5±7.80	318.3±8.78

^1^Data are given as means per treatment ± SEM, *n* = 36 at fasting (18 subject started with PUFA and 18 with placebo), *n* = 37 at the rest of the time points (19 subject started with PUFA and 18 with placebo) due to 2 and 1 subjects, respectively, performed the test incorrectly.

^2^ The total SA-test (test 1–4), *p* = 0.087 (ANOVA). The statistical calculations for the total SA-test is based on *n* = 36, the number of subjects that performed all four tests.

### Relations between cognitive performance and metabolic risk markers

The systolic blood pressure (F(1,36) = 4.56, *p* = 0.04, *d* = 0.46) and s-triglycerides (F(1,37) = 4.05, *p* = 0.05, *d* = 0.59) were significantly more suppressed after 5 weeks supplementation with omega-3 PUFA, compared with after the placebo (based on differences between after completion and prior to start of each intervention period). The results of the effects of n-3 PUFA on metabolic test markers are compiled in Table 4. As a general feature, the systolic blood pressure was inversely related to the performance in the cognitive tests. This relation was most pronounced in the WM-tests after the intervention with n-3 PUFA (WM 60 min: *r* = −0.35, *p* = 0.034, WM 110 min: *r* = −0.38, *p* = 0.022, WM 160 min (in the most difficult part): *r* = −0.36, *p* = 0.029). The fasting glucose concentrations were inversely related to the performance in the WM test at 110 min after n-3 PUFA (*r* = −0.32, *p* = 0.05). There was also a trend towards an inverse relation between f-glucose concentrations after the placebo period and WM test at 60 min (the most difficult part, *r* = −0.23, *p* = 0.069). Concentrations of triacylglycerides tended to be inversely related to cognitive performance. The strongest relation was seen in the WM-test at 60 min after start of the standardized breakfast following n-3 PUFA (*r* = −0.30, *p* = 0.066). Serum TNF-α concentrations were inversely related to the performance in the SA-test at fasting, after the placebo period (*r* = −0.33, *p* = 0.05).

**Table 4:** Results of the physiological test parameters before and after five weeks interventions with 3g/d omega-3 PUFA from fish oil and placebo, respectively

**Physiological parameters**	***Omega-3***			***Placebo***			
	Before	After	Δ-Omega-3^1^	Before	After	Δ- Placebo^2^	Δ^3^
Weigh (kg)	72.1±2.0	72.3±2.0	0.2±0.1	72.3±2.0	72.2±2.0	−0.1±0.1	ns
Systolic BP (mmhg)^4^	134±3	127±3	−7±2***	132±3	131±3	−1±2	P≤0.05
Diastolic BP (mmhg)^5^	79.2±1.4	76.9±1.4	−2.24±1.0*	78.8±1.2	77.3±1.4	−1.46±0.8	ns
f-Glucose (mmol/L, n=37)	5.4±0.1	5.5±0.1	0.1±0.1	5.5±0.1	5.4±0.1	−0.1±0.1	ns
ΔGlucose peak (mmol/L)	4.2±0.2	4.0±0.2	−0.2±0.2	4.3±0.2	4.2±0.2	−0.1±0.2	ns
Glucose 90 min IAUC (mmol*min/L)	217±13	213±11	−4±1	225±10	226±11	1±10	ns
Insulin (pmol/L)^6^	35±3	40±3	5±2*	37±3	43±04	6±3^**†**^	ns
FFA (mmol/L)^7^	0.28±0.02	0.26±0.02	−0.03±0.02	0.28±0.02	0.31±0.02	0.03±0.02	P=0.055
Triglycerides (mmol/L)^8^	1.63±0.10	1.45±0.09	−0.19±0.07*	1.58±0.10	1.66±0.11	0.08±0.07	P≤0.05
TNF-α (ng/L)	9.8±1.0	9.3±0.9	−0.48±0.44	8.9±0.8	8.5±0.8	−0.43±0.30	ns
Adiponectin (mg/L)	12±1	12±1	0±0	12±1	12±1	0±0	ns
Malondialdehyd (μmol/L)	2.0±0.1	2.0±0.1	0.1±0.1	1.9±0.1	2.0±0.1	0.1±0.1	ns

^1^ Changes (Δ) in test variables after 5 weeks PUFA supplementation compared with baseline (prior to start of PUFA). ^2^ Changes (Δ) in test variables after 5 weeks placebo supplementation compared with baseline (prior to start of placebo).

*: *p* < 0.05, ***: *p* < 0.001 (ANOVA) with respect to differences from baseline after 5 weeks intervention with PUFA.

^**†**^: *p* < 0.05 (ANOVA) with respect to differences from baseline after 5 weeks intake of the placebo.

^3^ P-values for differences between effects of PUFA (Δ-PUFA) and effects of placebo (Δ- placebo).

^4^ The systolic blood pressure was significantly more suppressed after 5 weeks supplementation with omega-3 PUFA compared with after 5 weeks with placebo [F(1,36) = 4.56, *p* = 0.04, *d* = 0.46]. The systolic BP was significantly lower after, compared to prior to the omega-3 period [F(1,36) = 14.70, *p* = 0.0005, *d* = 0.33]. n=37 in the BP analysis due to one participant failed to rest prior to one measurement.

^5^ The diastolic BP was significantly lower after, compared to prior to the omega-3 period [F(1,36) = 5.27, *p* = 0.028, *d* = 0.26]. There was a tendency toward lower diastolic BP after, compared with prior to the placebo period [F(1,36) = 3.31, *p* = 0.08, *d* = 0.19].

^6^ The s-insulin concentrations were significantly higher after, compared to prior to the omega-3 period [F(1,37) = 6,14, *p* = 0.018, *d* = 0.28] and placebo period [F(1,37) = 4.11, *p* = 0.050, *d* = 0.30], respectively.

^7^ There was a tendency towards concentrations of FFA to be more suppressed compared with after the placebo [F(1,37) = 3.94, *p* = 0.055, *d* = ], however, the concentrations of FFA after 5 weeks supplementation with omega-3 PUFA did not differ compared to prior omega-3 PUFA.

^8^The concentrations of s-triglycerides were significantly lower after, compared to prior to the omega-3 period [F(1,37) = 6.87, *p* = 0.013, *d* = 0.32], and significantly more suppressed after 5 weeks supplementation with omega-3 PUFA compared with after the placebo [F(1,37) = 4.05, *p* = 0.05, *d* = 0.59 ].

Abbreviations: ns: no significant differences between Δ-PUFA and Δ- placebo (ANOVA), f: fasting. P: plasma. S: serum. IAUC: incremental area under the curve. BP: blood pressure.

Values are mean±SEM. Statistical evaluations are performed with analysis of variance (ANOVA general linear model). N = 38, except for evaluation of BP, (n = 37).

Evaluations of relations between improvements in cognitive performance (WM-1) and lowering of triacylglycerides, or systolic blood pressure, were performed in subject groups showing lower triacylglyceride concentrations (n=23) or systolic blood pressure (n = 25) after n-3 PUFA compared with after placebo. Pearson correlations revealed a tendency towards a relation between improved performance (WM-1) and lowered systolic blood pressure(*r* = −0.37, *p* = 0.072), whereas no relations were detected between effects on triacylglycerides and improvement in WM-1.

## Discussion

The results show that daily intake of n-3 PUFA from fish oil during five weeks significantly improved cognitive functions (WM capacity) in healthy subjects. In addition there was a tendency towards better performance in the SA test after the n-3 PUFA period (SA-test no. 1–4, *p* = 0.087). DHA + EPA are involved in a number of brain functions that may modulate cognitive functions, e.g. neurotransmission and regulation of signal transduction pathways [1], and are also important structural components in neuronal cell membranes. In addition n-3 PUFA possesses several anti-inflammatory properties [36]. A growing body of data link chronic inflammation to poorer cognitive functions [37]. For example, in a middle-aged group of healthy subjects, circulating levels of IL-6 were inversely related to performance on a cluster of cognitive tests evaluating auditory recognition memory, attention, working memory, and executive function [37]. Interestingly, there was an inverse relation between TNF-α concentrations and performance in the SA-test in the present study. The relationship between inflammation and cognitive performance indicate that n-3 PUFA may be beneficial to cognitive functions due to a general anti-inflammatory effect; involving also effects on neuro-inflammation. Low grade chronic inflammation is increasingly also recognised as an important factor in the development of metabolic disorders such as diabetes type 2 [38] and cardiovascular disease [39] ( i.e. conditions that predispose for cognitive decline [14,40-42]).

In addition to improved cognitive performance, n-3 PUFA improved acknowledged cardiometabolic risk markers, i.e. systolic blood pressure and triglycerides. The systolic blood pressure was inversely related to performance on cognitive tests and there was also a tendency toward an inverse relation between cognitive performance and triglycerides (*P* = 0.066). The reductions in triglycerides and systolic blood pressure in the present cohort of healthy mature subjects were similar to those previously described in hyper-triglyceridaemic subjects after daily intake of 1g of fish- or seal oil for six weeks [20], highlighting the cardioprotective properties of n-3 PUF in healthy subjects.

The novelty of the present investigation is that it simultaneously evaluated effects of n-3 PUFA on cognition, as well as on cardiometabolic risk markers in healthy subjects. The relation between higher levels of cardiometabolic risk markers and inferior cognitive performance in healthy subjects, as observed in the present study, highlights the potential of a preventive dietary approach in the combat of both metabolic disorders and associated cognitive decline.

Available studies of effects of n-3 PUFA have mainly used different fatty acids as placebo. In the current study we included a non-oil based placebo product. The rationale for not choosing oil for placebo is that several fatty acids possess known or suggested metabolic and/or cognitive effects, and are therefore not inert to the test variables investigated in studies of metabolism and cognition [8,43-45]. A potential limitation of our study relates to the fact that the n-3 PUFA was administered in the form of a capsule, whereas the placebo treatment was in tablet form, since it was impossible to seal a capsule containing water. However, the test subjects were uninformed as to the activity of the PUFA and placebo supplement. It should also be noted that it is difficult to blind an intake of fish oil due to side effects such as ‘fishy burps’ [46]. An additional potential study limitation may be that no data is available concerning subjects’ blood- and/or red blood cell membrane phospholipid concentrations of n-3 PUFA.

## Conclusions

In conclusion, the present study reveals that five weeks daily intake of omega-3 PUFA from fish oil has the potential to improve cognitive functions and cardiometabolic risk factors in a healthy middle aged to elderly cohort. The relationship between outcome in cognitive tests and cardiometabolic risk factors highlights the importance of early dietary prevention to prevent cognitive decline secondary to cardiometabolic disorders. The dietary prevention strategy should preferably include fish in quantities to supply sufficient amounts of PUFA, in addition to other food groups with potential metabolic benefits e.g. whole grain, low-glycaemic index foods, fruits, berries, vegetables, and prebiotics [33,47-50]. Further studies are needed to clarify the underlying mechanism of the enhanced cognitive effect of omega-3 PUFA, and the relationship to cardiometabolic risk markers.

## Abbreviations

n-3 PUFA: Long chain n-3 polyunsaturated fatty acids; DHA: Docosahexaenoic acid; EPA: Eicosapentaenoic acid; BMI: Body mass index; WM: Working memory; SA: Selective attention.

## Competing interests

The authors declare that they have no competing interests.

## Authors’ contributions

Contributors: AN coordinate the study, was responsible for the study design, carried out the experimental work, the collection-, analysis and statistical calculations regarding the blood tests, and the evaluation and writing of the paper. KR and IS had the primary responsibility for the cognitive tests and the statistical analysis of cognitive test variables, and was involved in the evaluation and writing of the paper. IB was involved in the study design, and the evaluation, and writing of the paper. All authors read and approved the final manuscript.

## References

[B1] Dyall SC Michael-Titus AT Neurological benefits of omega-3 fatty acids Neuromolecular Med 2008 10 4 219 235 10.1007/s12017-008-8036-z 18543124

[B2] Ryan AS Astwood JD Gautier S Kuratko CN Nelson EB Salem N Jr Effects of long-chain polyunsaturated fatty acid supplementation on neurodevelopment in childhood: a review of human studies Prostaglandins Leukot Essent Fatty Acids 2010 82 4–6 305 314 20188533 10.1016/j.plefa.2010.02.007

[B3] Beydoun MA Kaufman JS Satia JA Rosamond W Folsom AR Plasma n-3 fatty acids and the risk of cognitive decline in older adults: the Atherosclerosis Risk in Communities Study Am J Clin Nutr 2007 85 4 1103 1111 17413112 10.1093/ajcn/85.4.1103

[B4] Dullemeijer C Durga J Brouwer IA van de Rest O Kok FJ Brummer RJ van Boxtel MP Verhoef P n 3 fatty acid proportions in plasma and cognitive performance in older adults Am J Clin Nutr 2007 86 5 1479 1485 17991662 10.1093/ajcn/86.5.1479

[B5] Dangour AD Allen E Elbourne D Fletcher A Richards M Uauy R Fish consumption and cognitive function among older people in the UK: baseline data from the OPAL study J Nutr Health Aging 2009 13 3 198 202 10.1007/s12603-009-0057-2 19262951 PMC12876379

[B6] Morris MC Evans DA Tangney CC Bienias JL Wilson RS Fish consumption and cognitive decline with age in a large community study Arch Neurol 2005 62 12 1849 1853 10.1001/archneur.62.12.noc50161 16216930

[B7] van Gelder BM Tijhuis M Kalmijn S Kromhout D Fish consumption, n-3 fatty acids, and subsequent 5-y cognitive decline in elderly men: the Zutphen Elderly Study Am J Clin Nutr 2007 85 4 1142 1147 17413117 10.1093/ajcn/85.4.1142

[B8] Eskelinen MH Ngandu T Helkala EL Tuomilehto J Nissinen A Soininen H Kivipelto M Fat intake at midlife and cognitive impairment later in life: a population-based CAIDE study Int J Geriatr Psychiatry 2008 23 7 741 747 10.1002/gps.1969 18188871

[B9] Nurk E Drevon CA Refsum H Solvoll K Vollset SE Nygard O Nygaard HA Engedal K Tell GS Smith AD Cognitive performance among the elderly and dietary fish intake: the Hordaland Health Study Am J Clin Nutr 2007 86 5 1470 1478 17991661 10.1093/ajcn/86.5.1470

[B10] van de Rest O Geleijnse JM Kok FJ van Staveren WA Dullemeijer C Olderikkert MG Beekman AT de Groot CP Effect of fish oil on cognitive performance in older subjects: a randomized, controlled trial Neurology 2008 71 6 430 438 10.1212/01.wnl.0000324268.45138.86 18678826

[B11] Yurko-Mauro K McCarthy D Rom D Nelson EB Ryan AS Blackwell A Salem N Jr Stedman M Beneficial effects of docosahexaenoic acid on cognition in age-related cognitive decline Alzheimers Dement 2010 6 6 456 464 10.1016/j.jalz.2010.01.013 20434961

[B12] Fontani G Corradeschi F Felici A Alfatti F Migliorini S Lodi L Cognitive and physiological effects of Omega-3 polyunsaturated fatty acid supplementation in healthy subjects Eur J Clin Invest 2005 35 11 691 699 10.1111/j.1365-2362.2005.01570.x 16269019

[B13] Antypa N Van der Does AJ Smelt AH Rogers RD Omega-3 fatty acids (fish-oil) and depression-related cognition in healthy volunteers J Psychopharmacol 2009 23 7 831 840 10.1177/0269881108092120 18583436

[B14] Ruis C Biessels GJ Gorter KJ van den Donk M Kappelle LJ Rutten GE Cognition in the early stage of type 2 diabetes Diabetes Care 2009 32 7 1261 1265 10.2337/dc08-2143 19366968 PMC2699741

[B15] Cavalieri M Ropele S Petrovic K Pluta-Fuerst A Homayoon N Enzinger C Grazer A Katschnig P Schwingenschuh P Berghold A Schmidt R Metabolic syndrome, brain magnetic resonance imaging, and cognition Diabetes Care 2010 33 12 2489 2495 10.2337/dc10-0851 20852031 PMC2992176

[B16] Lamport DJ Lawton CL Mansfield MW Dye L Impairments in glucose tolerance can have a negative impact on cognitive function: a systematic research review Neurosci Biobehav Rev 2009 33 3 394 413 10.1016/j.neubiorev.2008.10.008 19026680

[B17] van den Berg E Dekker JM Nijpels G Kessels RP Kappelle LJ de Haan EH Heine RJ Stehouwer CD Biessels GJ Cognitive functioning in elderly persons with type 2 diabetes and metabolic syndrome: the Hoorn study Dement Geriatr Cogn Disord 2008 26 3 261 269 10.1159/000160959 18841011

[B18] Raffaitin C Feart C Le Goff M Amieva H Helmer C Akbaraly TN Tzourio C Gin H Barberger-Gateau P Metabolic syndrome and cognitive decline in French elders: the Three-City Study Neurology 2011 76 6 518 525 10.1212/WNL.0b013e31820b7656 21288982

[B19] Hassenstab JJ Sweat V Bruehl H Convit A Metabolic syndrome is associated with learning and recall impairment in middle age Dement Geriatr Cogn Disord 2010 29 4 356 362 10.1159/000296071 20424454 PMC2889255

[B20] Meyer BJ Lane AE Mann NJ Comparison of seal oil to tuna oil on plasma lipid levels and blood pressure in hypertriglyceridaemic subjects Lipids 2009 44 9 827 835 10.1007/s11745-009-3333-3 19727884

[B21] Micallef MA Garg ML Anti-inflammatory and cardioprotective effects of n-3 polyunsaturated fatty acids and plant sterols in hyperlipidemic individuals Atherosclerosis 2009 204 2 476 482 10.1016/j.atherosclerosis.2008.09.020 18977480

[B22] Sartorelli DS Damiao R Chaim R Hirai A Gimeno SG Ferreira SR Dietary omega-3 fatty acid and omega-3: omega-6 fatty acid ratio predict improvement in glucose disturbances in Japanese Brazilians Nutrition 2010 26 2 184 192 10.1016/j.nut.2009.03.013 19647413

[B23] Navas-Carretero S Perez-Granados AM Schoppen S Vaquero MP An oily fish diet increases insulin sensitivity compared to a red meat diet in young iron-deficient women Br J Nutr 2009 102 4 546 553 10.1017/S0007114509220794 19210857

[B24] Radeborg K Briem V Hedman LR The effect of concurrent task difficalty on working memory during simulated driving Ergonomics 1999 42 767 777 10.1080/001401399185441

[B25] Daneman M Carpenter PA Individual differences in working memory and reading J Verbal Learning Verbal Behav 1980 19 450 466 10.1016/S0022-5371(80)90312-6

[B26] Kiewra KA Benton SL The relationship between information processing ability and notetaking Contemp Educ Psychol 1988 13 33 34 10.1016/0361-476X(88)90004-5

[B27] Engle RW Carullo JJ Collins KW Individual differences in working memory for comprehension and following directions J Educ Res 1991 84 253 262

[B28] Kyllonen PC Christal RE Reasoning ability is (little more than) working-memory capacity?! Intelligence 1990 14 389 433 10.1016/S0160-2896(05)80012-1

[B29] Kyllonen PC Stephens DL Cognitive abilities as determinants of success in aquiring logic skill Learn Individ Differ 1990 2 129 150 10.1016/1041-6080(90)90020-H

[B30] Kyllonen PC Dennis A Tapsfield P Is working memory capacity Spearman's g? Human abilities: their nature and measurement 1996 Erlbaum, Mahwah, NJ 49 75

[B31] Engle RW Kane MJ Tuholski SW Miake A, Shah P Individual differences in working memory capacity and what they tell us about controlled attention, general fluid intelligence, and functions of the prefrontal cortex Models of working memory: Mechanisms of active maintenance and executive controll 1999 Cambridge University Press, New York 102 134

[B32] Nilsson A Radeborg K Bjorck I Effects of differences in postprandial glycaemia on cognitive functions in healthy middle-aged subjects Eur J Clin Nutr 2009 63 1 113 120 10.1038/sj.ejcn.1602900 17851459

[B33] Nilsson AC Ostman EM Holst JJ Bjorck IM Including indigestible carbohydrates in the evening meal of healthy subjects improves glucose tolerance, lowers inflammatory markers, and increases satiety after a subsequent standardized breakfast J Nutr 2008 138 4 732 739 18356328 10.1093/jn/138.4.732

[B34] Chiou JF Hu ML Elevated lipid peroxidation and disturbed antioxidant enzyme activities in plasma and erythrocytes of patients with uterine cervicitis and myoma Clin Biochem 1999 32 3 189 192 10.1016/S0009-9120(98)00110-6 10383079

[B35] Cohen J A power primer Psychol Bull 1992 112 1 155 159 19565683 10.1037//0033-2909.112.1.155

[B36] Calder PC n-3 polyunsaturated fatty acids, inflammation, and inflammatory diseases Am J Clin Nutr 2006 83 6 Supp 1505S 1519S 16841861 10.1093/ajcn/83.6.1505S

[B37] Marsland AL Petersen KL Sathanoori R Muldoon MF Neumann SA Ryan C Flory JD Manuck SB Interleukin-6 covaries inversely with cognitive performance among middle-aged community volunteers Psychosom Med 2006 68 6 895 903 10.1097/01.psy.0000238451.22174.92 17132839

[B38] Bertoni AG Burke GL Owusu JA Carnethon MR Vaidya D Barr RG Jenny NS Ouyang P Rotter JI Inflammation and the incidence of type 2 diabetes: the Multi-Ethnic Study of Atherosclerosis (MESA) Diabetes Care 2010 33 4 804 810 10.2337/dc09-1679 20097779 PMC2845031

[B39] Conen D Ridker PM Clinical significance of high-sensitivity C-reactive protein in cardiovascular disease Biomark Med 2007 1 2 229 241 10.2217/17520363.1.2.229 20477398

[B40] Strachan MW Deary IJ Ewing FM Frier BM Is type II diabetes associated with an increased risk of cognitive dysfunction? A critical review of published studies Diabetes Care 1997 20 3 438 445 10.2337/diacare.20.3.438 9051402

[B41] Taylor VH MacQueen GM Cognitive dysfunction associated with metabolic syndrome Obes Rev 2007 8 5 409 418 10.1111/j.1467-789X.2007.00401.x 17716298

[B42] Yaffe K Weston AL Blackwell T Krueger KA The metabolic syndrome and development of cognitive impairment among older women Arch Neurol 2009 66 3 324 328 10.1001/archneurol.2008.566 19273750 PMC2685462

[B43] Segarra AB Ruiz-Sanz JI Ruiz-Larrea MB Ramirez-Sanchez M de Gasparo M Banegas I Martinez-Canamero M Vives F Prieto I The profile of Fatty acids in frontal cortex of rats depends on the type of fat used in the diet and correlates with neuropeptidase activities Horm Metab Res 2011 43 2 86 91 10.1055/s-0030-1269855 21120792

[B44] Solfrizzi V D'Introno A Colacicco AM Capurso C Del Parigi A Capurso S Gadaleta A Capurso A Panza F Dietary fatty acids intake: possible role in cognitive decline and dementia Exp Gerontol 2005 40 4 257 270 10.1016/j.exger.2005.01.001 15820606

[B45] Barcelo-Coblijn G Kitajka K Puskas LG Hogyes E Zvara A Hackler L Jr Farkas T Gene expression and molecular composition of phospholipids in rat brain in relation to dietary n-6 to n-3 fatty acid ratio Biochim Biophys Acta 2003 1632 1–3 72 79 12782153 10.1016/s1388-1981(03)00064-7

[B46] Mann NJ O'Connell SL Baldwin KM Singh I Meyer BJ Effects of seal oil and tuna-fish oil on platelet parameters and plasma lipid levels in healthy subjects Lipids 2010 45 8 669 681 10.1007/s11745-010-3450-z 20652432

[B47] McKeown NM Meigs JB Liu S Saltzman E Wilson PW Jacques PF Carbohydrate nutrition, insulin resistance, and the prevalence of the metabolic syndrome in the Framingham Offspring Cohort Diabetes Care 2004 27 2 538 546 10.2337/diacare.27.2.538 14747241

[B48] Hu FB Willett WC Optimal diets for prevention of coronary heart disease JAMA 2002 288 20 2569 2578 10.1001/jama.288.20.2569 12444864

[B49] McKeown NM Meigs JB Liu S Wilson PW Jacques PF Whole-grain intake is favorably associated with metabolic risk factors for type 2 diabetes and cardiovascular disease in the Framingham Offspring Study Am J Clin Nutr 2002 76 2 390 398 12145012 10.1093/ajcn/76.2.390

[B50] Rosen LA Silva LO Andersson UK Holm C Ostman EM Bjorck IM Endosperm and whole grain rye breads are characterized by low post-prandial insulin response and a beneficial blood glucose profile Nutr J 2009 8 42 10.1186/1475-2891-8-42 19781071 PMC2761418

